# Quantification of left atrial function in patients with non-obstructive hypertrophic cardiomyopathy by cardiovascular magnetic resonance feature tracking imaging: a feasibility and reproducibility study

**DOI:** 10.1186/s12968-019-0589-5

**Published:** 2020-01-02

**Authors:** Yingxia Yang, Gang Yin, Yong Jiang, Lei Song, Shihua Zhao, Minjie Lu

**Affiliations:** 1 Department of Magnetic Resonance Imaging, Fuwai Hospital and National Center for Cardiovascular Diseases, Chinese Academy of Medical Sciences and Peking Union Medical College, Beijing, 100037 China; 2 Department of Radiology, The People’s Hospital of Guangxi Zhuang Autonomous Region, Nanning, 530021 China; 3 Department of Echocardiography, Fuwai Hospital and National Center for Cardiovascular Diseases, Chinese Academy of Medical Sciences and Peking Union Medical College, Beijing, 100037 China; 4 Key Laboratory of Cardiovascular Imaging (Cultivation), Chinese Academy of Medical Sciences, Beijing, 100037 China; 5 Department of Cardiology, Fuwai Hospital and National Center for Cardiovascular Diseases, Chinese Academy of Medical Sciences and Peking Union Medical College, Beijing, 100037 China

**Keywords:** Hypertrophic cardiomyopathy, Cardiovascular magnetic resonance, Feature tracking, Left atrial dysfunction

## Abstract

**Background:**

Atrial fibrillation (AF) is the most common arrhythmia in hypertrophic cardiomyopathy (HCM) and is associated with adverse outcomes in HCM patients. Although the left atrial (LA) diameter has consistently been identified as a strong predictor of AF in HCM patients, the relationship between LA dysfunction and AF still remains unclear. The aim of this study is to evaluate the LA function in patients with non-obstructive HCM (NOHCM) utilizing cardiovascular magnetic resonance feature tracking (CMR-FT).

**Methods:**

Thirty-three patients with NOHCM and 28 healthy controls were studied. The global and regional LA function and left ventricular (LV) function were compared between the two groups. The following LA global functional parameters were quantitively analyzed: reservoir function (total ejection fraction [LA total EF], total strain [ε_s_], peak positive strain rate [SRs]), conduit function (passive ejection fraction [LA passive EF], passive strain [ε_e_], peak early-negative SR [SRe]), and booster pump function (active ejection fraction [LA active EF], active strain [ε_a_], peak late-negative SR [SRa]). The LA wall was automatically divided into 6 segments: anterior, antero-roof, inferior, septal, septal-roof and lateral. Three LA strain parameters (ε_s_, ε_e_, ε_a_) and their corresponding strain rate parameters (SRs, SRe, SRa) during the reservoir, conduit and booster pump LA phases were segmentally measured and analyzed.

**Results:**

The LA reservoir (LA total EF: 57.6 ± 8.2% vs. 63.9 ± 6.4%, *p* < 0.01; ε_s_: 35.0 ± 12.0% vs. 41.5 ± 11.2%, *p* = 0.03; SRs: 1.3 ± 0.4 s^− 1^ vs. 1.5 ± 0.4 s^− 1^, *p* = 0.02) and conduit function (LA passive EF: 28.7 ± 9.1% vs. 37.1 ± 10.0%, *p* < 0.01; ε_e_: 18.7 ± 7.9% vs. 25.9 ± 10.0%, *p* < 0.01; SRe: − 0.8 ± 0.3 s^− 1^ vs. -1.1 ± 0.4 s^− 1^, *p* < 0.01) were all impaired in patients with NOHCM when compared with healthy controls, while LA booster pump function was preserved. The LA segmental strain and strain rate analysis demonstrated that the ε_s_, ε_e_, SRe of inferior, SRs, SRe of septal-roof, and SRa of antero-roof walls (all *p* < 0.05) were all decreased in the NOHCM cohort. Correlations between LA functional parameters and LV conventional function and LA functional parameters and baseline parameters (age, body surface area and NYHA classification) were weak. The two strongest relations were between ε_s_ and LA total EF(*r* = 0.84, *p* < 0.01), ε_a_ and LA active EF (*r* = 0.83, *p* < 0.01).

**Conclusions:**

Compared with healthy controls, patients with NOHCM have LA reservoir and conduit dysfunction, and regional LA deformation before LA enlargement. CMR-FT identifies LA dysfunction and deformation at an early stage.

## Background

Hypertrophic cardiomyopathy (HCM) is the most common genetic heart disorder and has a prevalence of 1/200 people [1]. Patients with HCM usually have abnormal left atrial (LA) size and can develop atrial fibrillation (AF). Increased LA diameter has consistently been identified as a strong predictor of AF in HCM [2, 3]. Some studies have shown that enlargement of the LA results from left ventricular (LV) diastolic dysfunction [4, 5]. Elevated LV filling pressures from LV diastolic dysfunction are transmitted back to the LA, which results in LA enlargement and dysfunction [6]. The normal function of the LA is to fill the LV and can be separated into three portions: (1) reservoir function (collection of pulmonary venous blood during LV systole); (2) conduit function (passage of pulmonary venous blood flow to LV during early LV diastole); (3) booster pump function (augment LV filling during late LV diastole/atrial systole) [7].

HCM patients with obstructive hypertrophic cardiomyopathy (HOCM) tend to have associated LA enlargement and LA dysfunction likely from LV diastolic dysfunction [6]. However, the impact of LV hypertrophy on the LA function in non-obstructive HCM (NOHCM) remains unknown. Previous publications investigating LA function in HCM patients have primarily focused on the LA size [8] and volume [9]. However, these two parameters alone may be insufficient to describe the complexity of the LA function in HCM [10]. Thus, analysis of the LA functional parameters of strain and strain rate (SR) using non-invasive imaging modalities such as echocardiography speckle tracking (STE) and cardiovascular magnetic resonance (CMR) feature tracking (CMR-FT) have been proposed for further evaluation. STE has been proven to be a feasible and reproducible technique to evaluate LA function [11] and has demonstrated significant LA function impairment in HCM [12–14]. CMR-FT is a new quantitative method for wall motion assessment that is analogous to STE [15]. CMR-FT is acquired on the routine CMR cine images and has the advantages of higher spatial resolution, larger field of view and better reproducibility when compared to STE [16]. LA global and regional function in HCM patients have not yet been well studied by CMR-FT, especially in NOHCM. The aim of this study is to evaluate the feasibility and reproducibility of CMR-FT for the quantification of global and regional LA function in patients with NOHCM. In addition, we analyze the differences of global and regional LA function between NOHCM patients and healthy subjects to answer the question as to whether the alteration of LA function precedes LA enlargement.

## Methods

### Patient population

From January 2018 to June 2018, 33 consecutive NOHCM patients who met the inclusion criteria were enrolled [17]. The inclusion criteria included: (1) CMR demonstrated LV hypertrophy (maximal wall thickness ≥ 15 mm in an adult or ≥ 13 mm in an adult with relatives with HCM) in the absence of other diseases that could cause the LV hypertrophy [17], (2) left ventricular outflow tract (LVOT) gradient ≤30 mmHg on 2-dimensional echocardiography at rest or ≤ 50 mmHg during or immediately following exercise [17], (3) normal size of both ventricles and atria [18] and LV ejection fraction (EF) > 50%. Exclusion criteria included: (1) history of coronary artery disease, myocardial infarction or myocarditis, (2) history of septal myectomy or alcoholic septal ablation, (3) history of atrial fibrillation or atrial fibrillation at the time of CMR, (4) known contraindications to CMR imaging. Twenty-eight healthy subjects were selected as control group. This control group consisted of 13 females and 15 males with no history of cardiovascular disease, normal physical examination, normal electrodcardiogram (ECG) and echocardiography. Written informed consent was obtained from all study participants. This study was approved by our local institutional review boards.

### CMR imaging

CMR images were acquired using 3 T scanners (MR750, General Electric Healthcare, Waukesha, Wisconsin, USA or Ingenia, Philips Healthcare, Best, the Netherlands) with retrospective ECG gating and 8-channel cardiac coil. Subjects were examined in the supine position. Standard axial and sagittal dark blood images were performed using semi-Fourier single-shot sequence with the following parameters: slice thickness: 8 mm, gap: 4 mm, repetition time (TR): 2 heart beats, echo time (TE): 40 ms, matrix size: 224 × 192, field of view (FOV): 340 × 280 mm. The balanced steady-state free precession sequences (bSSFP) cine images included coverage of the entire LV and LA using short-axis slices, one 2-chamber view and one 4-chamber view with the following parameters: slice thickness: 8 mm, gap: 2 mm, TR: 2.9~3.4 ms, TE: 1.5~1.7 ms, matrix size: 192 × 224~224 × 256, FOV: 320 × 320 mm~ 380 × 380 mm, temporal resolution: 30~55 ms (dependent on heart rate) [19].

### CMR analysis

LV end-diastolic diameter (LVEDD) was measured on the short-axis cine image of the LV at the level of the papillary muscles. LV end-diastolic volume index (LVEDVi), LV end-systolic volume index (LVESVi), LV ejection fraction (LVEF), LV cardiac output (CO), LV cardiac index (CI) and LV mass (LVM) were measured using a post-processing workstation (Philips Intellispace Portal 7.0 and Advantage Workstation 4.6). LV endocardial and epicardial contours were drawn on LV short-axis cine images (papillary muscles were excluded).

**Fig. 1:**
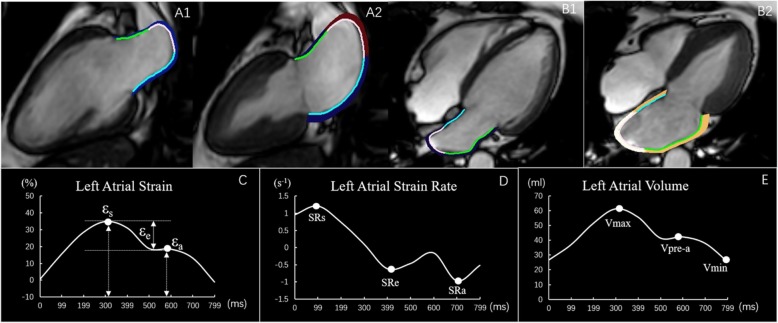
This figure shows a representative example of left atrial (LA) tracking on both the 2-and 4- chamber cines in a normal control subject. **A1** and **A2** left ventricular (LV) end-diastole and end-systole respectively on the 2-chamber view, **B1**and **B2** LV end-diastole and end-systole respectively on the 4-chamber view. **C** and **D** The LA strain and strain rate curves. The total strain (ε_s_), Passive strain (ε_e_) and active strain (ε_a_) were identified from the strain curves. The strain rates during LV systole (SRs), LV early diastole (SRe), and atrial contraction (SRa) were also determined from the strain rate curve. **E** LA volume curve. The LA maximum volume (Vmax), the pre-contraction volume (Vpre-a), and the minimum volume (Vmin) are shown here

LA anteroposterior (AP) diameters were measured on transversal dark blood images. LA volume and function were analyzed using commercial post-processing software (QStrain, Medis Suite 3.1, Leiden, the Netherlands). The LA endocardial border was manually delineated using a point-and-click approach when the atrium was at its maximum and minimum volumes in both the 2-and 4-chamber cine images (pulmonary veins and LA appendage were excluded) (Fig. 1A1-B2). Then the contours were automatically propagated in all frames throughout the entire cardiac cycle (25 frames/cardiac cycle). CMR-FT was visually reviewed to ensure accurate tracking. In cases of inadequate tracking, the endocardial border was manually readjusted and then the propagation algorithm was reapplied. LA global strain and SR were calculated as the average of the two and four chamber views [20]. Tracking was blindly repeated three times in both the 2-and 4-chamber views, and the results of the LA volume, strain and SR from the three tracking repetitions were averaged in both views. Three aspects of LA strain were analyzed as previously described [19–21] (Fig. 1C): total strain (ε_s_, corresponding to LA reservoir function), active strain (ε_a_, corresponding to LA booster pump function) and passive strain (ε_e_, corresponding to LA conduit function, the difference between ε_s_ and ε_a_). Accordingly, three SR parameters were evaluated (Fig. 1D): peak positive strain rate (SRs, corresponding to LA reservoir function), peak early negative strain rate (SRe, corresponding to LA conduit function) and peak late negative strain rate (SRa, corresponding to LA booster pump function).

LA volume (LAV) was assessed at LV end-systole (LAV_max_), at LV diastole before LA contraction (LAV_pre-a_), and at late LV diastole after LA contraction (LAV_min_) (Fig. 1E). The parameters of the LAV were obtained from a volume curve generated using Simpson’s method. From the LAV, the LA emptying fractions (LAEF) were calculated as follows: (1) LA total EF = (LAV_max_-LAV_min_) × 100%/LAV_max_, (2) LA passive EF = (LAV_max_-LAV_pre-a_) × 100%/LAV_max_, (3) LA active EF = (LAV_pre-a_-LAV_min_) × 100%/LAV_pre-a_ [21].

**Fig. 2:**
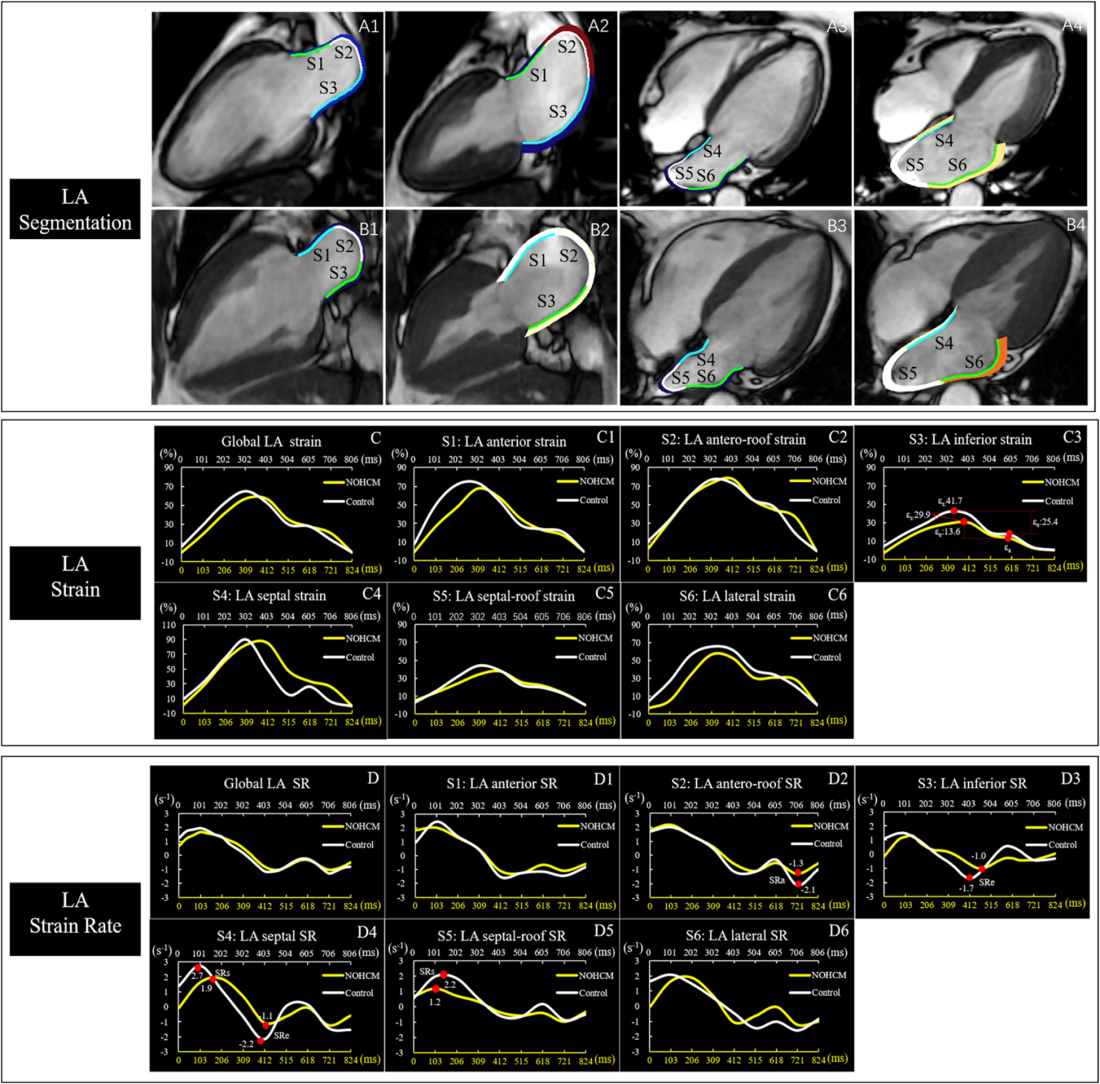
LA segmentation in representative cases of a healthy subject (**A1–4**) and a NOHCM patient (**B1–4**). The LA wall is automatically divided into 6 segments by the software [segment1(S1): anterior, segment2(S2): antero-roof, segment3(S3): inferior, segment4(S4): septal, segment5(S5): septal-roof, segment6(S6): lateral]. Comparison of LA global strain (**C**) and strain rate (**D**), segmental strain (**C1–6**) and strain rate (**D1–6**) between the non-obstructive hypertrophic cardiomyopathy (NOHCM) (yellow line) and the control (white line), the LA global strain and strain rate in the NOHCM were similar to the control, while segmental strain (inferior) and strain rate (antero-roof, inferior, septal and septal-roof) were lower in the NOHCM than the control. The yellow X axis represented the cardiac cycle length of a patient with NOHCM, and the white X axis represented the cardiac cycle length of a healthy control. ε_s_ = total strain, ε_e_ = passive strain, ε _=_, active strain, SRs = peak positive strain rat, SRe = peak early negative strain rate, SRa = peak late negative strain rate. Time dependent curves of the strain parameters were plotted offline using raw values provided by software

For estimating the LA segmental function, the software automatically divided the LA wall into 6 segments on both the 2- and 4-chamber views and generated strain curves and SR for each segment. As has been previously described [10], the LA segments were described as anterior, antero-roof, inferior, septal, septal-roof and lateral walls (Fig. 2A1-B4). The values of the LA segmental strain and SR were obtained from the average of the three repeated measurements. In the case of insufficient tracking quality, the corresponding segments were excluded from the final analysis. Patients with inadequate tracking quality in more than three segments were excluded from the study.

### Reproducibility

The intra- and inter-observer variability for the LA volume, strain and SR measurements were assessed by the coefficient of variation (CV), intraclass correlation coefficient (ICC) and Bland Altman analysis in 20 randomly selected subjects (10 healthy subjects and 10 NOHCM patients). Intra-observer reproducibility was established by the same observer who re-analyzed the same 20 subjects after 1 month. Inter-observer reproducibility was assessed by a second-independent observer, blinded to the first observer’s results.

### Statistical analysis

Continuous variables were presented as the means ± SD and categorical variables as frequencies or percentages. Comparison of the continuous variables between the two groups was performed by using independent *t* test for normally distributed data, Mann-Whitney *U* test for non-normally distributed data, and chi-square test for categorical variables. Pearson’s or Spearman’s correlation was performed to investigate the potential relationship between LV conventional parameters, baseline parameters and LA function. The correlation was considered weak if correlation coefficient was < 0.5, moderate if correlation coefficient was between 0.5–0.7, and strong if correlation coefficient was > 0.7 [22]. All statistical analyses were performed with SPSS (version 22.0, Statistical Package for the Social Sciences (SSPS), International Business Machines, Inc., Armonk, New York, USA) and MedCalc software (version 15.0, Mariakerke, Belgium). *P* value of 0.05 or less was considered significant.

## Results

### Basic demographic data

Of the 33 NOHCM patients, 23 were male (70%) and the average age was 40.3 ± 14.8 years (range: 19 to 72 years). The 28 healthy controls consisted of 15 men (53%) with an average age of 37.0 ± 11.0 years (range: 19 to 52 years). There were no significant differences in gender, age, body surface area (BSA), systolic blood pressure or diastolic blood pressure between the two groups (*p* > 0.05). Eight NOHCM patients were asymptomatic (New York Heart Association (NYHA) class I) and 25 NOHCM patients were symptomatic including 21 in NYHA class II, 3 in NYHA class III and 1 in NYHA class IV. The baseline demographic data of the NOHCM patients and the controls are summarized in Table 1.

**Table 1:** Patient demographic information

	NOHCM *n* = 33	Healthy control *n* = 28	*P-value*
Gender, male, n (%)	23 (70)	15 (53)	0.13
Age (years)	40.3 ± 14.8	37.0 ± 11.0	0.24
Body surface area (m^2^)	1.8 ± 0.2	1.9 ± 0.2	0.11
Systolic blood pressure (mmHg)	113.2 ± 11.9	115.5 ± 8.6	0.12
Diastolic blood pressure (mmHg)	73.8 ± 10.0	73.6 ± 8.7	0.42
Family history of HCM, n (%)	3 (9)	–	–
NYHA class			
I, n (%)	8 (24)	–	–
II, n (%)	21 (64)	–	–
III, n (%)	3 (9)	–	–
IV, n (%)	1 (3)	–	–
Chest pain, n (%)	7 (21)	–	–
Chest tightness, n (%)	19 (58)	–	–
Syncope, n (%)	3 (9)	–	–
Mitral regurgitation			
trace-mild, n (%)	3 (9)	–	–
mid/severe, n (%)	0 (0)	–	–

Data are expressed as mean ± SD, or as n (%). HCM, hypertrophic cardiomyopathy; NYHA, New York Heart Association

### LV conventional functional parameters

The LVEDD in the NOHCM patients was smaller than that of the normal controls (45.5 ± 5.1  vs. 47.8 ± 3.2 mm, *p* = 0.03). However, LVEF and LVM were greater (63.8 ± 5.8% vs. 59.3 ± 4.6% (*p*<0.01) and 103.9 ± 11.9  vs. 64.5 ± 17.5 g (*p* = 0.01), for NOHCM and healthy controls, respectively). There were no statistical differences in the LVEDVi, LVESVi, LVCO or LVCI between the two groups (all *p* > 0.05, Table 2).

**Table 2:** Comparison of left ventricular function between the NOHCM and control groups

	NOHCM *n* = 33	Healthy Control *n* = 28	*P-value*
LVEDD (mm)	45.5 ± 5.1	47.8 ± 3.2	0.03
LVEDVi (ml/m^2^)	62.6 ± 18.2	64.5 ± 14.1	0.64
LVESVi (ml/m^2^)	22.9 ± 8.9	26.3 ± 6.5	0.08
LVEF (%)	63.8 ± 5.8	59.3 ± 4.6	<0.01
LVCO (L/min)	5.2 ± 1.2	5.1 ± 1.1	0.59
LVCI (L/min/m^2^)	3.1 ± 0.7	2.9 ± 0.6	0.32
LVmass (g)	103.9 ± 11.9	64.5 ± 17.5	0.01

Data are expressed as mean ± SD

*LV* left ventricular, *EDD* end-diastolic diameter, *EDVi* end-diastolic volume index, *ESVi* end-systolic volume index, *EF* emptying fraction, *CO* cardiac output, *CI* cardiac index

### LA structure and function

The LA anteroposterior diameter index and the LA maximum volume index were not significantly different between the NOHCM patients and the healthy controls. However, the LA pre-contractile volume index and the minimum volume index were higher in the NOHCM patients (23.3 ± 6.2 vs. 19.7 ± 4.9 ml/m^2^ (*p* = 0.02) and 13.6 ± 3.5 vs. 11.3 ± 2.9 ml/m^2^ (*p* < 0.01), respectively). The LA reservoir and conduit function were both significantly lower in the NOHCM group (LA total EF: 57.6 ± 8.2% vs. 63.9 ± 6.4% (*p* < 0.01), ε_s_: 35.0 ± 12.0% vs. 41.5 ± 11.2% (*p* = 0.03), SRs: 1.3 ± 0.4  vs. 1.5 ± 0.4 s^− 1^ (*p* = 0.02), LA passive EF: 28.7 ± 9.1% vs. 37.1 ± 10.0% (*p* < 0.01), ε_e_: 18.7 ± 7.9% vs. 25.9 ± 10.0% (*p* < 0.01), SRe: − 0.8 ± 0.3 vs. -1.1 ± 0.4 s^− 1^ (*p* < 0.01)). In contrast, the LA booster pump function (including both the LA active EF, ε_a_ and the SRa) did not show a significant difference (*p* > 0.05 for all). Detailed measurements of the LA global function are shown in Table 3.

**Table 3:** Comparison of global left atrial function between the NOHCM and control groups

	NOHCM	Healthy control	*P-value*
	*n* = 33	*n* = 28	
LADI-AP (mm/m^2^)	14.4 ± 2.3	14.1 ± 2.5	0.85
LAVI (ml/m^2^)			
LAVImax	32.6 ± 7.9	31.1 ± 5.5	0.41
LAVIpre-a	23.3 ± 6.2	19.7 ± 4.9	0.02
LAVImin	13.6 ± 3.5	11.3 ± 2.9	< 0.01
Reservoir function			
LA total EF (%)	57.6 ± 8.2	63.9 ± 6.4	< 0.01
ε_s_ (%)	35.0 ± 12.0	41.5 ± 11.2	0.03
SRs (s^− 1^)	1.3 ± 0.4	1.5 ± 0.4	0.02
Conduit function			
LA passive EF (%)	28.7 ± 9.1	37.1 ± 10.0	< 0.01
ε_e_ (%)	18.7 ± 7.9	25.9 ± 10.0	< 0.01
SRe (s^−1^)	−0.8 ± 0.3	− 1.1 ± 0.4	< 0.01
Booster pump function			
LA active EF (%)	40.3 ± 10.1	42.1 ± 9.2	0.49
ε_a_ (%)	16.3 ± 6.5	15.6 ± 6.3	0.67
SRa (s^−1^)	− 1.1 ± 0.5	−1.0 ± 0.4	0.49

Data are expressed as mean ± SD

*LADI-AP* left atrial anteroposterior diameter index, *LAEF* left atrial emptying fraction, *LAVI*_*max*_ left atrial maximum volume index, *LAVI*_*pre-a*_ left atrial pre-atrial contraction volume index, *LAVI*_*min*_ left atrial minimum volume index, *ε*_*s*_ total strain, *ε*_*e*_ passive strain, *ε*_*a*_ active strain, *SRs* peak positive strain rate, *SRe* peak early negative strain rate, *SRa* peak late negative strain rate

Segmental strain analysis was successfully performed on 366 segments. ε_s_, ε_e_, SRe of the inferior wall as well as the SRs, SRe of septal-roof wall were lower in the NOCHM (*p* < 0.05). Although the global booster LA pump function was not different between the two groups, further segmental analysis showed that the SRa of the antero-roof wall was significantly decreased in NOHCM (*p* < 0.01). Detailed LA segmental functional parameters are summarized in Table 4.

**Table 4:** Comparison of regional left atrial function between the NOHCM and control groups

	NOHCM	Healthy control	*P-value*
	*n* = 33	*n* = 28	
ε_s_ (%)			
Anterior	41.4 ± 26.4	48.0 ± 20.7	0.27
Antero-roof	50.6 ± 23.2	46.5 ± 19.3	0.57
Inferior	29.7 ± 19.9	47.9 ± 18.9	< 0.01
Septal	28.0 ± 21.5	34.1 ± 16.8	0.11
Septal-roof	30.5 ± 19.6	37.3 ± 13.4	0.22
Lateral	37.3 ± 22.1	48.0 ± 19.9	0.17
ε_e_ (%)			
Anterior	20.0 ± 13.6	25.3 ± 14.5	0.13
Antero-roof	19.8 ± 14.6	19.4 ± 10.4	0.64
Inferior	14.7 ± 18.1	28.1 ± 18.7	< 0.01
Septal	15.8 ± 15.1	19.3 ± 11.5	0.11
Septal-roof	16.1 ± 11.1	21.4 ± 8.3	0.10
Lateral	19.7 ± 15.2	27.5 ± 15.1	0.30
ε_a_ (%)			
Anterior	21.4 ± 14.7	22.8 ± 13.0	0.71
Antero-roof	30.9 ± 11.6	27.1 ± 12.9	0.23
Inferior	15.0 ± 11.9	19.8 ± 10.9	0.09
Septal	12.2 ± 9.9	14.9 ± 12.6	0.37
Septal-roof	14.4 ± 13.5	15.9 ± 11.6	0.51
Lateral	17.6 ± 13.4	20.5 ± 11.6	0.56
SRs (s^−1^)			
Anterior	1.5 ± 0.9	1.7 ± 0.7	0.23
Antero-roof	1.7 ± 0.7	1.6 ± 0.8	0.61
Inferior	1.3 ± 0.6	1.6 ± 0.7	0.08
Septal	1.1 ± 0.6	1.2 ± 0.4	0.5
Septal-roof	1.1 ± 0.6	1.4 ± 0.6	0.03
Lateral	1.3 ± 0.6	1.4 ± 0.5	0.49
SRe (s^−1^)			
Anterior	−0.8 ± 0.5	−1.0 ± 0.5	0.09
Antero-roof	−1.1 ± 0.7	−0.9 ± 0.6	0.28
Inferior	− 0.7 ± 0.6	−1.0 ± 0.6	0.04
Septal	− 0.8 ± 0.4	− 0.9 ± 0.5	0.18
Septal-roof	−0.6 ± 0.3	−1.1 ± 0.5	< 0.01
Lateral	−0.8 ± 0.6	−1.2 ± 0.7	0.07
SRa (s^−1^)			
Anterior	−1.1 ± 0.8	−1.2 ± 0.6	0.85
Antero-roof	−0.9 ± 0.6	−1.4 ± 0.7	< 0.01
Inferior	−1.1 ± 0.7	−1.0 ± 0.8	0.53
Septal	−0.9 ± 0.4	−0.6 ± 0.7	0.08
Septal-roof	−0.8 ± 0.6	−0.9 ± 0.6	0.94
Lateral	−1.2 ± 0.7	− 1.1 ± 0.5	0.61

Data are expressed as mean ± SD

*ε*_*s*_ total strain, *ε*_*e*_ passive strain, *ε*_*a*_ active strain, *SRs* peak positive strain rate, *SRe* peak early negative strain rate, *SRa* peak late negative strain rate

### Correlation between LV conventional function and LA function in NOHCM patients

**Fig. 3:**
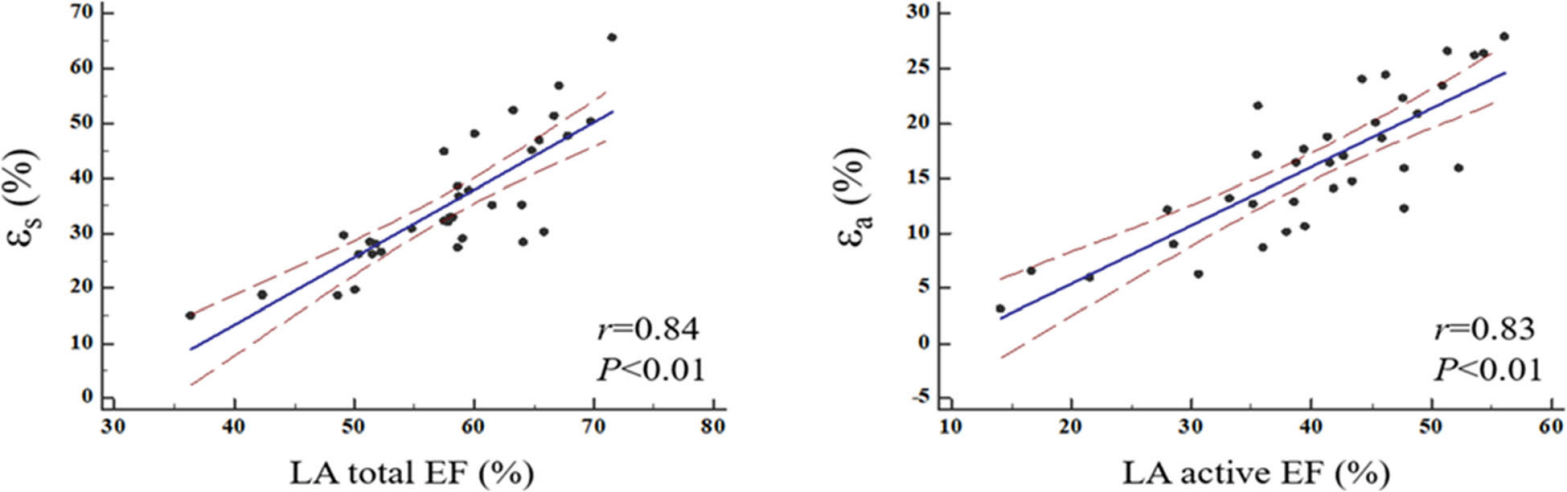
Scatter plots showing correlations of LA total emptying fraction (EF) and ε_s_, LA active EF and ε_a_. ε_s_ = total strain, ε_a_ = active strain

There were weak correlations between the LV functional parameters (LVEDD, LVEDVi, LVESVi, CI, LVM and LVEF), baseline parameters (age, BSA, NYHA class) and the LA functional components (EF, strain and SR) (Table 5). The correlation between ε_s_ and LA total EF and the correlation between ε_a_ and the LA active EF were the two strongest correlations (*r* = 0.84, *p* < 0.001; *r* = 0.83, *p* < 0.001; respectively) (Fig. 3).

**Table 5:** Correlation of LV conventional function and LA function in the NOHCM group

	LATEF	LAPEF	LAAEF	ε_s_	ε_e_	ε_a_	SRs	SRe	SRa
Age	−0.10	−0.47^**^	0.28	0.21	−0.02	0.40	0.16	−0.22	−0.28
BSA	0.03	0.01	0.05	0.07	0.01	0.11	−0.02	−0.03	0.18
LVEDD	0.19	0.05	0.14	0.14	0.16	0.07	0.14	−0.03	0.18
LVEF	0.26	0.17	0.18	0.18	0.07	0.24	0.20	−0.22	−0.28
LVEDVi	0.09	0.29	−0.18	0.07	0.24	−0.15	−0.09	0.07	0.15
LVESVi	−0.07	0.06	−0.19	−0.06	0.09	−0.22	− 0.24	0.18	0.19
LVCI	0.18	0.34	−0.11	0.08	0.16	−0.04	0.18	−0.20	0.01
LVM	−0.39^*^	−0.17	− 0.31	−0.30	− 0.31	−0.17	− 0.26	0.32	0.27
LATEF	–	–	–	0.84^**^	0.76^**^	0.62^**^	0.63^**^	−0.61^**^	−0.48^**^
LAPEF	–	–	–	0.35^*^	0.61^**^	−0.09	0.23	−0.35^*^	−0.29
LAAEF	–	–	–	0.70^**^	0.38^*^	0.83^**^	0.52^**^	−0.40^*^	−0.27
NYHA class	−0.42^*^	−0.31	− 0.23	−0.40^*^	− 0.37^*^	−0.24	− 0.37^*^	0.08	0.05

Data represents the correlation coefficients. ^*^p<0.05, ^**^p<0.01

*BSA* body surface area, *LV* left ventricular, *EDD* end-diastolic diameter, *EF* emptying fraction, *EDVi* end-diastolic volume index, *ESVi* end-systolic volume index, *CI* cardiac index, *LA* left atrial, *TEF* total emptying fraction, *PEF* passive emptying fraction, *AEF* active emptying fraction, *ε*_*s*_ total strain, *ε*_*e*_ passive strain, *ε*_*a*_ active strain, *SRs* peak positive strain rate, *SRe* peak early negative strain rate, *SRa* peak late negative strain rate, *LVM* left ventricular mass

### Reproducibility

**Fig. 4:**
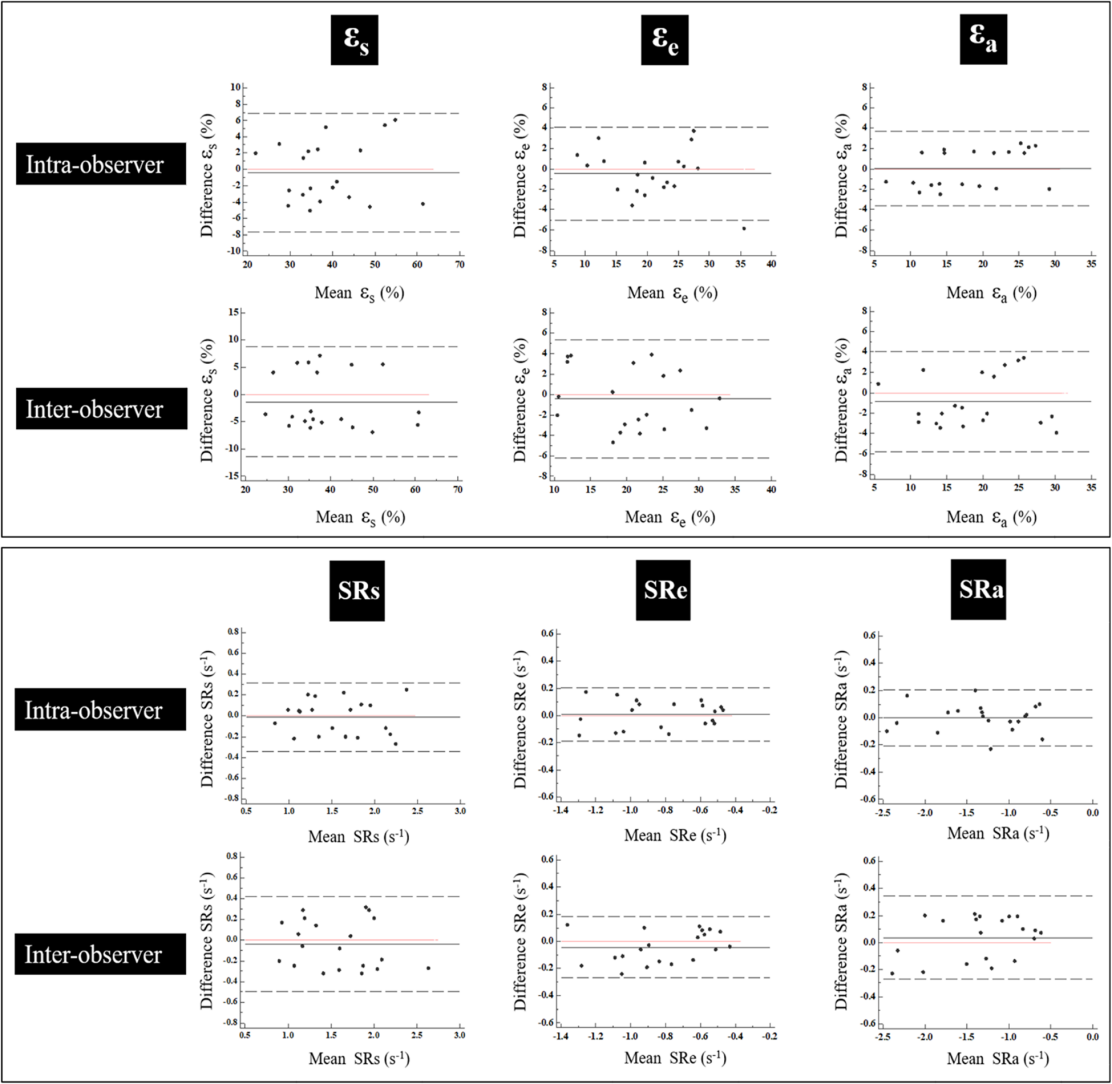
Bland Altman Plots for intra- and inter-observer variability. Bland Altman Plots for intra-and inter-observer variability obtained for global LA strain and SR. ε_s_ = total strain, ε_e_ = passive strain, ε_a_ = active strain, SRs = peak positive strain rate, SRe = peak early negative strain rate, SRa = peak late negative strain rate

Global LA strain and SR parameters were reproducible on an intra- and inter-observer level. Bland-Altman Plots for global strain and SR measurements are shown in Fig. 4. The CV% and ICC for the LA global functional parameters are summarized in Table 6. Global strain and the SR assessment were much more reproducible than the regional, segmental analysis. For intra-observer reproducibility, the segmental measurement of the inferior wall of SRa was the highest in terms of reproducibility: ICC 0.94 (0.79–0.99). For inter-observer reproducibility, the septal wall SRe had the highest reproducibility: ICC 0.88 (0.59–0.97). The least reproducible segmental measurement for both intra-and inter-observer reproducibility was the anterior ε_e_: 0.43 (− 0.23–0.81) and 0.42 (− 0.24–0.82), respectively.

**Table 6:** Intra-observer and inter-observer reproducibility for LA functional parameters

	Intra-observer			Inter-observer		
	CV (%)	ICC	95% CI	CV (%)	ICC	95% CI
LAVmax	6.5	0.95	0.87–0.98	7.4	0.93	0.83–0.97
LAVpre-a	7.1	0.95	0.88–0.98	8.6	0.93	0.82–0.97
LAVmin	6.7	0.96	0.90–0.98	7.7	0.95	0.87–0.98
LA total EF	2.2	0.95	0.89–0.98	2.7	0.93	0.82–0.97
LA passive EF	6.6	0.94	0.85–0.97	8.4	0.89	0.73–0.95
LA active EF	4.3	0.96	0.89–0.98	6.6	0.88	0.72–0.95
ε_s_	6.6	0.93	0.83–0.97	9.3	0.88	0.72–0.95
ε_e_	7.9	0.94	0.86–0.98	10.0	0.91	0.76–0.96
ε_a_	7.1	0.96	0.90–0.98	9.8	0.93	0.84–0.97
SRs	7.4	0.93	0.84–0.97	10.3	0.88	0.73–0.95
SRe	8.4	0.94	0.86–0.98	10.6	0.91	0.79–0.96
SRa	5.5	0.98	0.96–0.99	8.4	0.96	0.89–0.98

*CV* coefficient of variation, *ICC* intraclass correlation coefficient, *LAV*_*max*_ left atrial maximum volume, *LAV*_*pre-a*_ left atrial pre-atrial contraction volume, *LAV*_*min*_ left atrial minimum volume, *LAEF* left atrial emptying fraction, *ε*_*s*_ total strain, *ε*_*e*_ passive strain, *ε*_*a*_ active strain, *SRs* peak positive strain rate, *SRe* peak early negative strain rate, *SRa* peak late negative strain rate

## Discussion

To the best of our knowledge, this is the first study evaluating both global and regional LA function by CMR-FT in NOHCM patients. This study elucidated several findings: CMR-FT is a promising method for quantification of the LA function in patients with NOHCM, LA global and regional reservoir dysfunction as well as LA conduit dysfunction was observed in NOHCM patients before LA dilation, the LA booster pump function did not show any significant difference between the patients with NOHCM and healthy control group, and regional function in the antero-roof LA wall was significantly decreased in NOHCM patients.

LA remodeling consists of both structural and functional changes. In HCM patients, LA structural remodeling has been shown to be related to LV diastolic dysfunction, degree of mitral regurgitation, and LVOT obstruction [23]. LA enlargement has been proven to positively predict the risk of AF [24]. Several studies have demonstrated that increased LA size in HCM patients is associated with impaired LA function [25–27]. The incidence of AF in HCM patients is reported to be up to 20% of HCM patients (the annual incidence of AF is 2% per year) and has a poor prognosis [28, 29]. The findings of our study show that LA dysfunction occurs prior to LA enlargement in NOHCM patients and thus suggests a relationship between LA dysfunction and AF occurrence in HCM patients prior to enlargement of the LA [26]. However, it should be noted that the although the LA size of the enrolled NOHCM patients in our study was normal and not significantly different than our normal control group, it was larger than healthy controls in previous studies [13, 23].

According to the results of our study, both LA reservoir and conduit dysfunction were observed NOHCM, which is consistent with findings of prior studies [21, 30]. LA diastole (corresponding to the reservoir function) depends on both the atrial compliance and the LV base descent during LV systole [31]. In patients with HCM, LA compliance is decreased due to increased wall stiffness caused by myocardial fibrosis [23, 28, 32]. Additionally, Liu et al. previously demonstrated that decreased LV global longitudinal strain in HCM patients may reduce the systolic movement of the LV basal wall [33], which would also decrease LA compliance and result in LA reservoir dysfunction. The LA conduit dysfunction is closely related to impaired LV compliance which is caused by a markedly thickened LV wall that often contains areas of myocardial fibrosis [8, 34]. Thus, our study further validated previous studies by also detecting LA reservoir and conduit dysfunction in this group of NOHCM patients. However, in contrast to the Kowallick’s study, the LA booster pump function in our study was not statistically different between the NOHCM patients and the normal controls. The LA booster pump functions in previous HCM patient studies in the literature has not been consistent and has been separately reported as normal [30], increased [21] or reduced [12]. LA contraction is influenced by pulmonary venous return (atrial pre-load), LV end-diastolic pressure (atrial after-load), and LV systolic reserve [8]. This discrepancy between our study and Kowallick’s study may be due to patient selection criteria of current study: no LA enlargement, no LV systolic dysfunction and no LVOT obstruction.

Previous studies have investigated the global LA strain and SR in HCM patients. Our study took this one step further and also analyzed the segmental strain and SR. We found that the function of inferior, septal-roof and antero-roof were regionally abnormal. The LA inferior wall dysfunction was probably related to the gravity of blood which causes compression of the inferior wall as well as related to the LA conduction system. The LA conduction system is associated with a line of conduction block from the LA roof to the inferior wall. This line corresponds to a change in subendocardial fiber orientation. Subendocardial fibers located on the septal side of the line have a longitudinal orientation. Subendocardial fibers located lateral to this line are oblique and circumferential in orientation [35]. The LA roof is fixed to the mediastinum by the pulmonary veins, which may account for the decreased LA roof wall function [34]. As a result, although the global function of SRa was preserved in the NOHCM patients, there was regional deformation dysfunction. The regional deformation detected by the SR analysis could help monitor the changes in LA function (Fig. 2).

We found no significant correlations between the LA functional parameters and conventional LV functional parameters. One possible reason for this may be that LV conventional functional parameters mainly reflect the LV systolic function. However, the LA function is more related to LV diastolic function instead of LV systolic function [36], which is exactly what our volumetric findings showed. Although LA maximal volume was similar to the normal controls, the volume of LA pre-contraction and after contraction were larger than those of the normal controls. Since the LA is directly exposed to the LV diastolic pressure during these two phases, the LA volume of these two phases could serve as an index in the assessment LV diastolic function. Furthermore, the majority of LA deformation parameters were significantly associated with LAEF, which might suggest potential correlation between LA wall deformation and LA size.

### Limitations

Several limitations of our study should be acknowledged. Weonly calculated the LA strain and SR. Further investigation of additional parameters such as displacement and movement speed are required in future studies. Also, this is a single center with a relatively modest sample size. Thus, subgroup analysis for types of HCM such as asymmetric hypertrophy of interventricular septum and apical hypertrophy were not performed. Because of its invasiveness, cardiac catheterization was not performed and thus cardiac physiological parameters including atrial and ventricular pressure were not obtained.

## Conclusions

CMR-FT technique is a reliable tool for quantitative assessment of LA function (volumetric and deformation parameters). Using CMR-FT, we found LA reservoir and conduit dysfunction occurs *prior* to LA enlargement in NOHCM patients. This suggests a relationship between LA dysfunction and AF risk in these patients. However, booster pump function in the NOHCM patients was not significantly different than normal controls. Additionally, deformation of the LA, in particular decreased regional function in the antero-roof LA wall, was also observed in the NOHCM patients. This LA regional deformation analysis may provide insight in assessing the LA performance over time in HCM patients.

## Data Availability

The datasets used and/or analyzed during the current study are available from the corresponding author on reasonable request**.**

## Abbreviations

AF: Atrial fibrillation
AP: Anterior-posterior
BSA: Body surface area
bSSFP: Balanced steady-state free precession sequences
CI: Cardiac index
CMR: Cardiovascular magnetic resonance
CO: Cardiac output
CV: Coefficient of variation
ECG: Electrocardiogram
EF: Ejection fraction
FOV: Field of view
FT: Feature tracking
HCM: Hypertrophic cardiomyopathy
HOCM: Obstructive hypertrophic cardiomyopathy
ICC: Intraclass correlation coefficient
LA: Left atrium/left atrial
LAEF: Left atrial emptying fraction
LAV: Left atrial volume
LV: Left ventricle/left ventricular
LVEDD: Left ventricular end-diastolic diameter
LVEDVi: Left ventricular end-diastolic volume index
LVESVi: Left ventricular end-systolic volume index
LVM: Left ventricular mass
LVOT: Left ventricular outflow tract
NOHCM: Non-obstructive hypertrophic cardiomyopathy
NYHA: New York Heart Association
SR: Strain rate
SRa: Peak late-negative strain rate
SRe: Peak early-negative strain rate
SRs: Peak positive strain rate
STE: Echocardiography speckle tracking
TE: Echo time
TR: Repetition time
ε_a_: Active strain
ε_e_: Passive strain
ε_s_: Total strain
